# We need to include bystander first aid in trauma research

**DOI:** 10.1186/s13049-017-0372-2

**Published:** 2017-03-23

**Authors:** Håkon Kvåle Bakke, Torben Wisborg

**Affiliations:** 1 Mo i Rana Hospital, Helgeland Hospital Trust, Mo i Rana, Norway; 2 Anaesthesia and Critical Care Research Group, Faculty of Health Sciences, IKM, University of Tromsø, Tromsø, Norway; 3 Department of Anaesthesiology and Intensive Care, University Hospital of North Norway, Tromsø, Norway; 4 Department of Anaesthesiology and Intensive Care, Hammerfest Hospital, Finnmark Health Trust, Hammerfest, Norway; 5 Norwegian National Advisory Unit on Trauma, Division of Emergencies and Critical Care, Oslo University Hospital, Oslo, Norway

## Abstract

**Background:**

The chain of trauma survival is a concept that originated in the area of out-of-hospital cardiac arrest (OHCA) and was adapted to the treatment of trauma. In out-of-hospital cardiac arrest research into bystander first aid has resulted in improved outcome. Whereas, in trauma research the first link of the chain of survival is almost ignored.

**Methods:**

In OHCA, cardiopulmonary resuscitation (CPR) from bystanders has been subject of a vast amount of research, as well as measures and programs to raise the rate of bystander CPR to cardiac arrest victims. These efforts have resulted in improved survival. The research effort has been well grounded in the research community, as demonstrated by its natural inclusion in the uniform reporting template (Utstein) for the treatment of OHCA. In trauma the bystander may contribute by providing an open airway, staunch bleedings, or prevent hypothermia. In trauma however, while the chain of survival has been adopted along with it distinct links, including bystander first aid, the consensus-based uniform reporting template for trauma (the Utstein template) does not include the bystander first aid efforts. There is extremely little research on what first aid measures bystanders provide to trauma victims, and on what impact such measures have on outcome. An important step to improve research on bystander first aid in trauma would be to include this as part of the uniform reporting template for trauma

**Conclusion:**

The lack of research on bystander first aid makes the first link in the trauma chain of survival the weakest link. We, the trauma research community, should either improve our research and knowledge in this area, or remove the link from the chain of survival

## Background

**Fig. 1:**
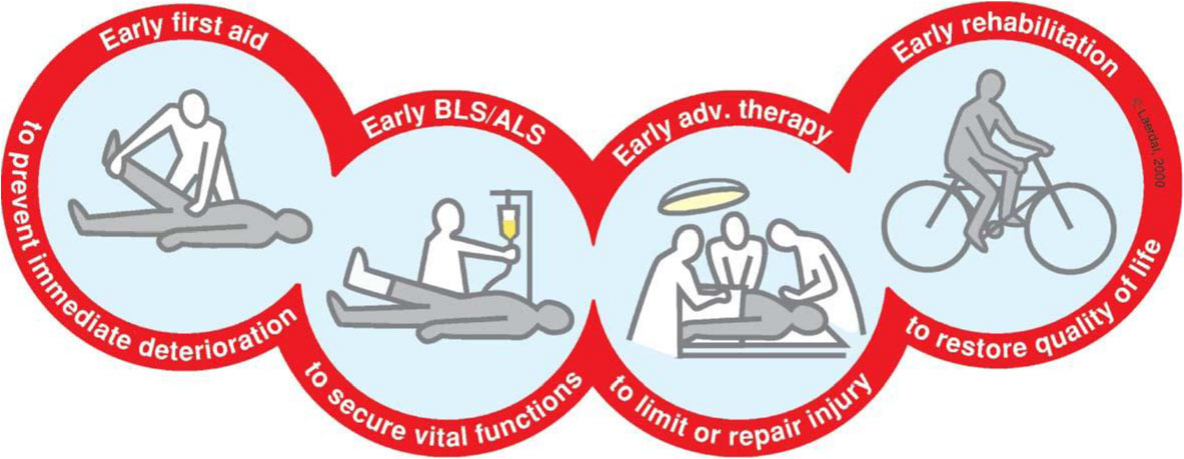
The trauma chain of survival. Reproduced with permission from Laerdal Medical, Stavanger, Norway

The chain of trauma survival is a concept that originated in the area of out-of-hospital cardiac arrest (OHCA) and adapted to the treatment of trauma [1, 2], (Fig. 1). It is boldly stated that each link in the chain is important for the survival of the trauma patient. Allegedly the chain is no stronger than its weakest link. This strong rhetoric, though fetching, belies the fact that we in trauma ignore the first link except in schematic presentations as Fig. 1.

In OHCA, cardiopulmonary resuscitation (CPR) from bystanders has been subject of a vast amount of research, as well as measures and programs to raise the rate of bystander CPR to cardiac arrest victims [3–5]. It is established that bystander CPR improves survival, and approximately how much [6, 7]. We have an overview of bystander CPR-rates and that they vary geographically [8, 9]. In addition to research into how CPR best can be carried out to improve patients survival [10] there is considerable attention on how best to teach CPR [11, 12] and if training has any effect [13, 14], as well as how dispatch instruction can improve quality and rate of bystander CPR [15, 16]. The research effort has been well grounded in the research community, as demonstrated by its natural inclusion in the uniform reporting template (Utstein) for the treatment of OHCA [17]. These investigative efforts have proven fruitful on several occasions, improving CPR-rate considerably, and even survival [9, 18].

In trauma bystander CPR has a more limited role, as traumatic cardiac arrest has a different aetiology and prognosis. However there are several other measures the bystander may provide, such as airway manoeuvres, compression of visible bleeding, and the prevention of hypothermia [19]. In trauma however, while the chain of survival has been adopted along with it distinct links, including bystander first aid, the consensus-based uniform reporting template (the Utstein template) does not bother to include the bystander first aid efforts [20]. There may be several reasons for this, but there is no attempt to explain the omission, so we are left to speculate. The current edition of the Utstein template has been criticised for omitting those trauma victims that die prehospitally, leading to inconsistency of methodology and terminology in the reporting on prehospital trauma deaths [21]. We fear that the lack of emphasis on prehospital care in the Utstein template is contributory to the lack of research on bystander first aid in trauma.

Because unfortunately, the omission of bystander first aid is not restricted to the reporting template for trauma. In 2012 we did a review on bystanders first aid in trauma and found a mere 11 studies world-wide on the subject, most were questionnaire-based surveys [19]. Little has changed since then, and a quick search in the PubMed database reveals that the only study that have investigated the role of bystander first aid is a study we ourselves conducted [22]. Likewise, in our work with dispatch assisted first aid we again found that this topic was well-covered for OHCA, but not for trauma. If we really do believe that the first aid from bystanders constitute an important link in the chain of trauma survival, then we should also conduct research concerning that link. In fact, the omission of this link from the Utstein template might have led researchers to the false belief that the question was already solved.

It is possible that bystander first aid in trauma has little effect on survival, that it is already optimised, and it is possible that our efforts on improving trauma survival is best spent elsewhere. But the fact of the matter is that we do not know, and we cannot know, until we have investigated. Reporting on bystander first aid should be included in the next Utstein template revision, and research should be encouraged and demanded. It may be that not all research will or can follow the template reporting recommendation. However, it will signal that research into first aid from bystanders actually is of interest, and we believe it is an important step to improving research in this field.

## Conclusion

The lack of research on bystander first aid makes the first link in the trauma chain of survival the weakest link. We, the trauma research community, should improve our research and knowledge in this area. Else we might as well remove this link from the chain entirely, not because we know whether it is unimportant, but because we have decided that we do not care.

## Abbreviations

CPR: Cardiopulmonary resuscitation
OHCA: Out-of-hospital cardiac arrest
